# Safety of surgical denervation of the common hepatic artery in insulin‐resistant dogs

**DOI:** 10.14814/phy2.14805

**Published:** 2021-03-26

**Authors:** Guillaume Kraft, Melanie Scott, Eric Allen, Dale S. Edgerton, Ben Farmer, Bobak R. Azamian, Alan D. Cherrington

**Affiliations:** ^1^ Department of Molecular Physiology and Biophysics Vanderbilt University School of Medicine Nashville TN USA; ^2^ Hormone Assay and Analytical Services Core Vanderbilt University Medical Center Nashville TN USA; ^3^ Metavention Eden Prairie MN USA

## Abstract

The objective of this study was to assess the safety of surgical common hepatic artery denervation (CHADN). This procedure has previously been shown to improve glucose tolerance in dogs fed a high‐fat high‐fructose (HFHF) diet. We assessed the hypoglycemic response of dogs by infusing insulin at a constant rate (1.5 mU/kg/min) for 3 h and monitoring glucose and the counterregulatory hormones (glucagon, catecholamine, and cortisol). After an initial hypoglycemic study, the dogs were randomly assigned to a SHAM surgery (n = 4) or hepatic sympathetic denervation (CHADN, n = 5) and three follow‐up studies were performed every month up to 3 months after the surgery. The level of norepinephrine (NE) in the liver and the pancreas was significantly reduced in the CHADN dogs, showing a decrease in sympathetic tone to the splanchnic organs. There was no evidence of any defect of the response to hypoglycemia after the CHADN surgery. Indeed, the extent of hypoglycemia was similar in the SHAM and CHADN groups (~45 mg/dl) for the same amount of circulating insulin (~50 µU/ml) regardless of time or surgery. Moreover the responses of the counterregulatory hormones were similar in extent and pattern during the 3 h of hypoglycemic challenge. Circulating lactate, glycerol, free fatty acids, and beta‐hydroxybutyrate were also unaffected by CHADN during fasting conditions or during the hypoglycemia. There were no other notable surgery‐induced changes over time in nutrients, minerals, and hormones clinically measured in the dogs nor in the blood pressure and heart rate of the animals. The data suggest that the ablation of the sympathetic nerve connected to the splanchnic bed is not required for a normal counterregulatory response to insulin‐induced hypoglycemia and that CHADN could be a safe new therapeutic intervention to improve glycemic control in individuals with metabolic syndrome or type 2 diabetes.

## 1 INTRODUCTION

Recent animal studies and clinical research suggest that modulation of the autonomic system might be able to counteract the effects of the metabolic syndrome (Akinseye et al., 2020; Guarino et al., 2017; Mahfoud et al., 2011; Maron et al., 2020). Specifically, common hepatic artery denervation (CHADN) significantly improved the glucose excursion during an oral glucose tolerance test in dogs with diet‐induced glucose intolerance (Kraft et al., 2019). The success of CHADN and other metabolic surgeries in improving glucose intolerance, however, raises the question of whether eliminating sympathetic nervous input to the liver alters hepatic responsiveness to insulin or predisposes to low blood sugar leading to a greater risk of iatrogenic hypoglycemia. The data in this paper come from a study that assessed efficacy and safety, the former was already published (Kraft et al., 2019). Given the ability of the procedure to reverse glucose intolerance caused by high‐fat high‐fructose (HFHF) feeding, it is essential to show that the procedure is also safe.

The usual response to hypoglycemia is mediated in part by the sympathetic system (tachycardia, sweating, and increase in blood pressure) (Cryer, 2011; Perin et al., 2001). Further, the release of key counterregulatory hormones is also under the control of the central nervous system (CNS). When the common hepatic artery is surgically denervated, the sympathetic tone to the liver is decreased, but as we recently showed sympathetic tone to the pancreas is also partially decreased (as evident by the lower norepinephrine (NE) content in the pancreas) (Kraft et al., 2019). It remains unclear what effect CHADN has on the response of the α cell, the CNS, and the liver in response to a hypoglycemic challenge.

The present paper contains data relating to the safety of CHADN as it relates to insulin‐induced hypoglycemia in diet‐induced glucose‐intolerant dogs. We studied the efficacy and safety of the technique in parallel, thereby reducing the number of dogs required. The efficacy data have been published (Kraft et al., 2019) and the safety data are presented here.

## 2 METHODS

These studies were conducted using nine male mongrel dogs weighing between 20 and 25 kg on entry into the study. The data relating to efficacy of the CHADN surgery on the postprandial response were published previously (Kraft et al., 2019). The dogs were housed in a facility that met American Association for Accreditation of Laboratory Animal Care guidelines, and the protocol was approved by the Vanderbilt University Medical Center Institutional Animal Care and Use Committee. The animals were fed a HFHF diet in which 22% of the energy was derived from protein, 52% from fat, and 26% from carbohydrate, the majority of which (17% of the total energy in the diet) was derived from fructose (TestDiet; PMI Nutrition, St. Louis, MO).

### 2.1 Protocol design

**FIGURE 1:**
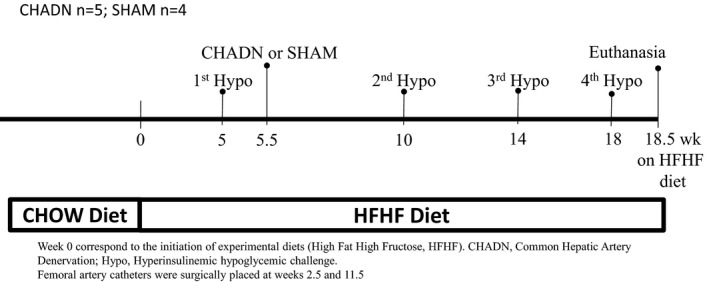
Experimental timeline

For each of the nine dogs, a series of hyperinsulinemic hypoglycemic clamps (Hypo) were performed. After 2.5 weeks of HFHF feeding, a femoral artery catheter was implanted under general anesthesia as described elsewhere (Coate et al., 2010). After 5 weeks of HFHF, and prior to denervation, a Hypo study was performed to assess the basal (pre‐treatment, first Hypo) response of the animals (Figure 1). A laparotomy was performed after 5.5 weeks of HFHF feeding and animals were randomly assigned to a hepatic surgical sympathectomized group (CHADN, n = 5) or sham surgery group (SHAM, n = 4). Follow‐up Hypo studies were performed 4.5, 8.5, and 12.5 weeks post‐CHADN or SHAM surgery (second, third, and fourth Hypo; Figure 1). After the second Hypo study, each animal underwent a minor surgery for the insertion of a new femoral artery catheter in the other back leg. The hepatic sympathetic denervation of the CHA was performed by stripping a 5 cm long area around the CHA and removing the visible nerve fibers encasing the vessel. All nerves surrounding the portal vein and its branches, as well as the vagus nerve, were left intact. Proper denervation was confirmed through measurement of organ‐specific tissue NE at the end of each protocol. The dogs were euthanized after an overnight fast at 13 weeks post‐surgery (18.5 weeks on diet) for tissue harvest. About 1–2 g of tissue was harvested from each lobe of the liver, freeze clamped in liquid nitrogen, and stored at −80°C until subsequent NE analysis. In addition, the duodenal, body and splenic lobes of the pancreas, a 2 cm segment of the duodenum immediately distal to the major duodenal papilla, and a piece of the pylorus were also harvested for NE analysis. Briefly, prior to analysis, 300 mg of tissue was homogenized in a PCA buffer containing glutathione. The homogenate was analyzed for NE by HPLC with electrochemical detection as described elsewhere (Goldstein et al., 1981). Glycogen and triglycerides (TG) were measured in the three largest liver lobes, that is, left medial, left lateral, and right medial, as described previously (Coate et al., 2010).

### 2.2 Experimental design

On the day of each Hypo study, the femoral artery catheter was exteriorized from its subcutaneous pockets using local anesthesia and additional intravenous catheters were placed in saphenous leg veins for infusion purposes. The dogs were placed in a Pavlov harness and allowed to rest for 60 min. A 30‐min control period was followed and blood was collected for analysis of glucose, insulin, glucagon, catecholamines, cortisol, lactate, alanine, glycerol, and free fatty acids, as described elsewhere (Coate et al., 2010; Kraft et al., 2019). Insulin was then infused into a leg vein (1.5 mU/kg/min) for 3 h. Every 30 min blood was drawn for analyte measurement. Plasma glucose was monitored every 5 min and glucose was infused as needed to prevent a drop below 40 mg/dl.

During the study, arterial blood pressure and heart rate were monitored using a Bridge Amp instrument (ADInstruments Inc, Colorado Springs, CO).

### 2.3 Animals

During the first week of HFHF feeding (ad libitum), the dogs ate ≈3500 kcal/day (compared to 2200 kcal/day before they were put on HFHF diet). Caloric consumption decreased by ≈15% after 1 week on the diet, eventually stabilizing at ≈2300 kcal/day. At that point, food consumption was not different between the SHAM and the CHADN groups.

Before the start of the diet, the animals' body weights were 22.8 ± 1.4 and 23.1 ± 1.2 kg in the SHAM and CHADN groups, respectively. After 1 week on the HFHF diet, the animals had gained 1.6 ± 0.5 and 2.1 ± 0.9 kg, respectively, then, by the time of the first post‐surgery (4.5 weeks) Hypo study, the weights were 26.0 ± 1.8 and 26.4 ± 1.6 kg in the SHAM and CHADN groups, respectively. Even at the end of the protocol (13 weeks after denervation surgery), no differences were observed between groups (25.8 ± 1.9 and 27.0 ± 0.6 kg in the SHAM and CHADN groups, respectively).

### 2.4 Fasting parameters

Blood was drawn before enrollment in the protocol and before each Hypo study, to measure a panel of analytes (Antech Labs, Southaven, MS) to monitor the animals' health and the function of the liver (AST, ALT, Alk Phos, total bilirubin, GGT, cholesterol, and proteins), the kidneys (BUN, creatinine, phosphorus, amylase, and albumin), the pancreas (glucose, amylase, lipase, and triglyceride) as well as the muscles and bones (calcium and phosphorus CPK and AST), and to monitor electrolytes (sodium, potassium, chloride, calcium, and phosphorous).

### 2.5 Data analysis

Data are expressed as means ± SD. Individual data and medians are shown after calculation of ΔAUC where possible. Two‐way ANOVA with repeated measures design was used (SigmaStat, Richmond, CA), with post‐hoc analysis performed using the Student–Newman–Keuls multiple comparisons model, with time and treatment as co‐factors. A *p* < 0.05 was considered significant.

## 3 RESULTS

There were no clinical complications in any animal throughout the study. Likewise, there were no histologic or hematologic abnormalities noted on autopsy. Analysis of liver NE content (Table 1) at the end of the study revealed virtually complete denervation of all liver lobes in the CHADN animals. The caudate and right lateral lobes had a small amount of measureable NE (~5% of the level in the SHAM dogs) in four of five animals. These data indicate that the sympathetic denervation of the liver remained virtually complete over the 3‐month study period (i.e., re‐innervation did not occur). Interestingly, hepatic denervation was associated with a decrease in the total NE content of the pancreas (53 ± 13% relative to SHAM animals). The splenic lobe of the pancreas was not denervated, whereas the duodenal lobe was denervated to some extent in all CHADN animals (range of 40%–92% NE reduction), and the body of the pancreas was denervated in all dogs by 90% or more. Duodenal NE content was reduced by an average of 78 ± 18% in the CHADN compared to SHAM group (the sections were taken 1–2 cm proximal to the entry of the pancreatic duct). The pylorus was substantially denervated in two of five dogs but not in the other three. Thus, complete surgical sympathetic denervation of the liver was associated with partial sympathetic denervation of the duodenum, pancreas, and, in some cases, the pylorus.

**TABLE 1:** Tissue norepinephrine content in SHAM and animals that underwent a common hepatic artery denervation (CHADN) at the end of each protocol

Tissue	Lobe	Norepinephrine ng/g tissue	
		SHAM (n = 4)	CHADN (n = 5)
Liver	Left central	393 ± 75	7 ± 4*
	Left Lateral	316 ± 68	3 ± 1*
	Left posterior	547 ± 56	4 ± 2*
	Quadrate	510 ± 148	26 ± 14*
	Right lateral	658 ± 211	15 ± 5*
	Right central	373 ± 67	3 ± 1*
Pancreas	Duodenal	496 ± 60	157 ± 55*
	Body	588 ± 114	85 ± 53*
	Splenic	692 ± 103	756 ± 170
Duodenum		243 ± 60	66 ± 25*
Pyloris		284 ± 69	133 ± 50

Values are means ± SD in ng/g of tissue.

Statistical analysis (*t* test) was performed.

* *p* < 0.05.

### 3.1 Fasting parameters

The results for baseline blood values can be found in the Tables S1 and S2 for the CHADN and SHAM groups, respectively. The effect of the HFHF was similar and minimal on these parameters in the two groups (week 0 vs. 5 results). No parameters increased or decreased meaningfully, with the cholesterol being the largest change. Not surprisingly, while on the diet, it increased by 25% and 38% in the CHADN and SHAM groups, respectively, but those values remained in the normal range and were not significantly different from their value at week 0.

The magnesium in both groups was slightly low after the start of the diet but this reduction was mild and has been previously described in animal models of obesity and other states of chronic inflammation (Nielsen, 2010). The supply of minerals in the diets (chow diet and HFHF) was the same, and more importantly, the change was not exacerbated or lessened by the experimental surgery performed. CHADN generated no consistent effect on any of the measured parameters over the course of the study.

### 3.2 Hyperinsulinemic hypoglycemic clamp (Hypo)

**FIGURE 2:**
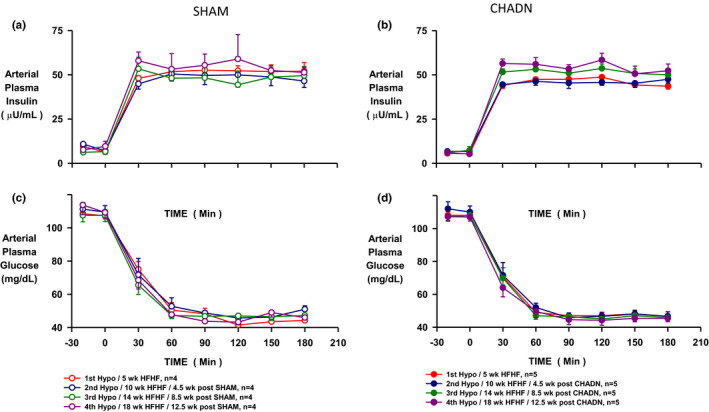
Arterial plasma insulin (a and b) and glucose (c and d) during successive hypoglycemia challenge in dog receiving a SHAM surgery (SHAM n = 4, open symbols) or a common hepatic denervation (CHADN, n = 5, solid symbols). The first Hypo was performed after the animals were fed a high‐fat high‐fructose diet for 5 weeks (in red). After that experiment, the animals were randomly assigned to the SHAM or CHADN group. Follow‐up Hypo studies were performed 4.5, 8.5, and 12.5 weeks later (in blue, green, and purple, respectively)

The insulin levels during the control period of each Hypo study were similar (~12 µU/ml) over time (from week 5 to week 18) and between groups (SHAM and CHADN). Likewise, the insulin level during the experimental period was similar (~50 µU/ml) over time (weeks 5–18) and between groups (SHAM and CHADN) (Figure 2a,b). The ΔAUC for the rise in insulin was not different between the SHAM and CHADN groups prior to or after surgery, and the ΔAUC did not change over time in either groups (Figure S1a,b). Neither the rate of fall of plasma glucose, the time to nadir (2 h), nor the glucose nadir itself (~45 mg/dl) were different between study weeks or study groups (Figure 2c,d). Consequently, the ΔAUC for the fall in glucose was not significantly affected by surgery or time (Figure S1c,d).

**FIGURE 3:**
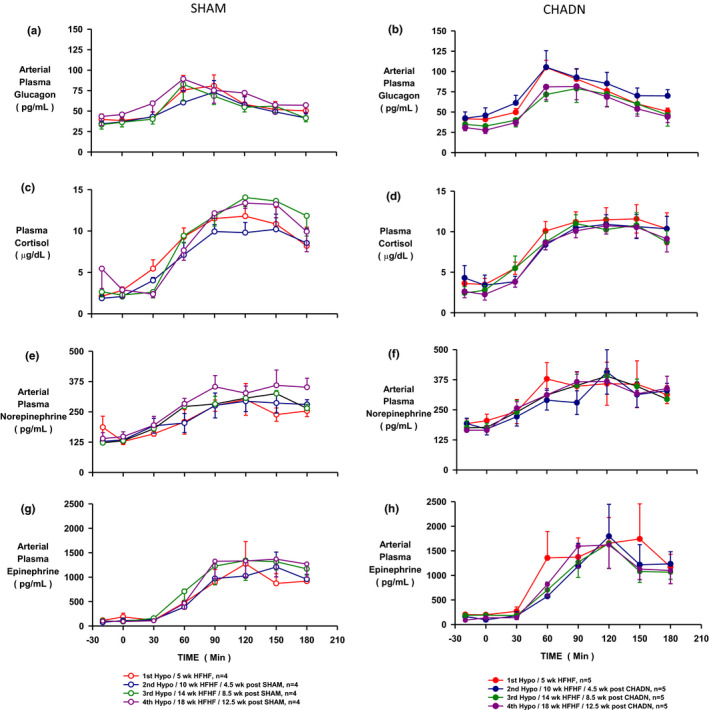
Arterial plasma glucagon (a and b), cortisol (c and d), norepinephrine (e and f), and epinephrine (g and h) during successive hypoglycemia challenge in dog receiving a SHAM surgery (SHAM n = 4, open symbols) or a common hepatic denervation (CHADN, n = 5, solid symbols). The first Hypo was performed after the animals were fed a high‐fat high‐fructose diet for 5 weeks and before the surgical intervention (in red). After that experiment, the animals were randomly assigned to the SHAM or CHADN group. Follow‐up Hypo studies were performed 4.5, 8.5, and 12.5 weeks later (in blue, green, and purple, respectively)

The control period plasma glucagon level was not different (~30 pg/ml) in either group (SHAM and CHADN) or between weeks of treatment (5–18 weeks). Hypoglycemia caused glucagon rise (~75–105 pg/ml) and peak between 30 and 60 min (Figure 3a,b) in all animals regardless of the surgery. The glucagon ΔAUCs during hypoglycemia were not different over the study weeks or between the study groups (Figure S1e,f). The plasma cortisol concentrations were similar and basal in both groups across time (5–18 weeks). They rose over the first 120 min of the hypoglycemic period and then plateaued at a value about 3‐fold basal for 30 min then fell slightly in both groups (Figure 3c,d, Figure S1g,h). The hypoglycemia‐driven increments in plasma NE and epinephrine (Figure 3e–h) peaked at about 60 min of the hypoglycemic period (~2‐fold and 20‐fold increased, respectively) and then fell slightly. There was no difference in NE or epinephrine response between groups or over time (Figure S1i–l). The counterregulatory hormonal responses to hypoglycemia were thus not different between SHAM and CHADN groups prior to surgery nor were they different between the two groups after surgery. These data collectively indicate that the hormonal responses to insulin‐induced hypoglycemia were unaffected by the interruption of sympathetic nerves to the liver and a partial reduction in sympathetic input to pancreas. As a result, the fall in plasma glucose was virtually identical prior to and up to 3 months after CHADN. It should be noted that it was not necessary to infuse glucose to limit hypoglycemia in any of the experiments.

### 3.3 Metabolic response to hypoglycemia

Insulin‐induced hypoglycemia is associated with an epinephrine‐driven rise in muscle glycogen breakdown resulting in increased muscle lactate production and a rise in blood lactate. During the pre‐surgery hypo‐challenge control period (first Hypo), blood lactate levels were not different in the two groups and rose significantly in response to hypoglycemia in both groups (Table 2). Insulin‐induced hypoglycemia is also associated with a neurally driven stimulation of lipolysis. The glycerol level, a good index of lipolysis, was twice basal during the last hour of the hypoglycemia in both groups. Neither the control period levels, nor the extent of increase in glycerol were significantly different in the SHAM and CHADN groups in response to the first Hypo (Table 2). Control period plasma free fatty acid (FFA) concentrations were similar in the SHAM and CHADN dogs. The increase of FFA observed during the first Hypo tended to be bigger in the SHAM group than in the CHADN group but the values were not significantly different. As the insulin level rose, and hypoglycemia developed, blood beta‐hydroxybutyrate (βOHB) levels initially fell in parallel with the fall in FFA, and then rose slightly, once again paralleling FFA. The baseline βOHB levels were higher in CHADN compared to the SHAM prior to the first Hypo challenge. In addition, the SHAM group showed an overall increase in βOHB level by the end of the Hypo period, whereas the CHADN group showed a significant decrease in the concentration of βOHB, which resulted in non‐significant difference in SHAM versus CHADN groups. Because these differences existed prior to surgical intervention, they are due to individual animal variability, not to an effect of CHADN.

**TABLE 2:** Arterial concentration of lactate, glycerol, free fatty acid (FFA) and betahydroxybutyrate (βOHB) in dogs during fasting conditions or at the end of a 3‐h insulin induced hypoglycemic challenge. A series of 4 hypoglycemia challenge were performed 4 weeks apart in animals being fed a high fat high fructose diet. The 1st Hypo was performed as a control before a surgical procedure was performed on the CHADN group (n = 5) to denervate the common hepatic artery whereas the SHAM group (n = 4) received a sham procedure

	Before treatment		After treatment (SHAM or CHADN)					
	1st Hypo		2nd Hypo		3rd Hypo		4th Hypo	
	SHAM	CHADN	SHAM	CHADN	SHAM	CHADN	SHAM	CHADN
Lactate								
Fasting	200.0 ± 16	233.0 ± 17	171.0 ± 16	254.2 ± 27*	182.3 ± 19	222.2 ± 23	175.7 ± 23	219.2 ± 14
Last hour of Hypo	321.5 ± 73^†^	589.6 ± 111^†^	314.4 ± 77^†^	479.6 ± 114^†^	367.6 ± 96^†^	475.1 ± 103^†^	311.4 ± 79^†^	413.4 ± 95^†^
Glycerol								
Fasting	80.9 ± 7	101.5 ± 10	64.5 ± 9	94.7 ± 9*	87.5 ± 6	109.1 ± 20	64.7 ± 6	104.6 ± 9*
Last hour of Hypo	183.4 ± 15^†^	189.3 ± 21^†^	129.6 ± 19^†^	195.2 ± 13^†^	185.8 ± 18^†^	197.9 ± 20^†^	165.3 ± 31^†^	199.7 ± 11^†^
FFA								
Fasting	868.3 ± 55	987.9 ± 56	898.4 ± 82	1100.3 ± 75	821.1 ± 66	1017.2 ± 105	825.8 ± 47	941.5 ± 109
Last hour of Hypo	1187.5 ± 104	1053.4 ± 128	1094.3 ± 134	1183.2 ± 64	1103.6 ± 156	1114.8 ± 87	1111.5 ± 52	1050.4 ± 94
βOHB								
Fasting	72.4 ± 10	142.4 ± 15	64.5 ± 6	127.0 ± 10	85.6 ± 11	118.5 ± 23	73.2 ± 17	114.0 ± 18
Last hour of Hypo	88.1 ± 13	76.2 ± 8	70.0 ± 14	84.2 ± 9	93.9 ± 21	91.2 ± 15	81.1 ± 16	84.8 ± 9

Data are expressed as means ± SD. Multiple way ANOVA was performed to test the effect of the treatment, the iteration of Hypo and the fasting versus hypoglycemia.

* *p* < 0.05 for CHADN versus SHAM at this Hypo iteration.

† *p* < 0.05 for Last hour of Hypo versus Fasting.

In the present study, neither the basal lactate level nor the hypoglycemia‐induced rise in blood lactate (~2‐fold) were altered significantly by CHADN regardless of number of weeks post‐surgery (Table 2). Likewise, CHADN had no effect on either the control period glycerol level or the hypoglycemia‐driven rise in glycerol regardless of the post‐surgical duration. Control period plasma FFA levels also were not altered by CHADN, and neither was the hypoglycemic‐induced rise in FFA levels. Thus, based on the glycerol and FFA data, sympathetic denervation of the liver did not impair the neural drive to fat caused by hypoglycemia. The baseline and hypoglycemia‐induced changes in blood βOHB levels were indistinguishable in the CHADN dogs prior to and after hepatic sympathetic denervation (Table 2). Thus, severing the common hepatic artery sympathetic nerves was without effect on ketogenesis during insulin‐induced hypoglycemia.

**FIGURE 4:**
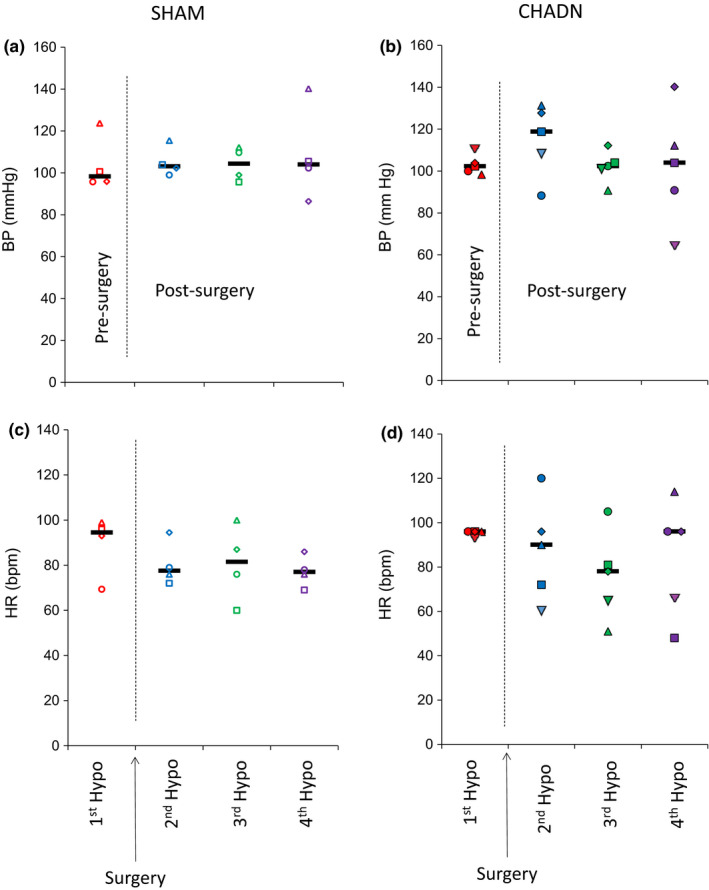
Blood pressure (BP, a and b) and heart rate (HR, c and d) in dogs in successive hypoglycemic challenge in dog receiving a SHAM surgery (SHAM n = 4, open symbols) or a common hepatic denervation (CHADN, n = 5, solid symbols). The first Hypo was performed after the animals were fed a high‐fat high‐fructose diet for 5 weeks and before the surgical intervention (in red). After that experiment, the animals were randomly assigned to the SHAM or CHADN group. Follow‐up Hypo studies were performed 4.5, 8.5, and 12.5 weeks later (in blue, green, and purple, respectively). Each symbol represent one individual animal studied multiple times, the black bars represent the median of the group

Figure 4 shows the mean arterial blood pressure (BP) and the heart rate (HR) in the control period of the insulin infusion experiments in the SHAM and CHADN dogs. Blood pressure (108 ± 4 and 106 ± 4 mm Hg) and heart rate (85 ± 8 and 86 ± 12 bpm) were not significantly different in the SHAM and CHADN groups, respectively. Likewise, neither hepatic denervation nor SHAM surgery altered those parameters at 4.5, 8.5, or 12.5 weeks post‐surgery.

The average terminal glycogen levels were 44 ± 4 and 51 ± 6 mg/g liver in the SHAM and CHADN animals, respectively, at the end of the 18.5 weeks of protocol, values consistent with normal hepatic glycogen levels after an overnight fast (ref). Likewise, liver TG levels were normal and there was no difference in TGs between groups (1.6 ± 0.9 vs. 1.8 ± 0.8 mg/g in SHAM vs. CHADN, respectively).

## 4 DISCUSSION

Although modulation of the autonomic nervous system has shown promise in the treatment of multiple metabolic conditions including hypertension and glycemic control, the safety of CHADN in particular has not been carefully evaluated. This study investigated the effect of sympathetic denervation of the liver on the iatrogenic insulin‐induced counterregulatory response to hypoglycemia. We found that the hormonal and metabolic responses are still intact following CHADN, allowing a normal response to insulin‐induced hypoglycemia. With similar hyperinsulinemia in both the CHADN and SHAM groups, the hypoglycemic nadir (≈45 mg/dl) was also similar. Hepatic denervation caused no impairment in the hypoglycemia‐induced elevations in plasma glucagon, cortisol, NE, or epinephrine and consequently, it had no effect on the observed hypoglycemia. Thus, our results argue for the safety of CHADN for the treatment of insulin resistance.

The HFHF‐fed dog model has been used widely to study glucose intolerance. Our goal here was to take dogs which had developed this defect and examine the impact of CHADN versus SHAM to improve glucose metabolism (Kraft et al., 2019), and at the same time examine the impact of CHADN versus SHAM on the response to hypoglycemia. However, the present study did not investigate the effect of HFHF on the hypoglycemic response compared to animals fed chow. After 4 weeks of a HFHF diet, our canine model presented a reduction in insulin sensitivity during a hyperglycemic hyperinsulinemic clamp (Coate et al., 2010), and it was unclear if this translated into a change in the counterregulatory response to hypoglycemia. In an unpublished study, the rate of glucose decrease was slower in animals fed HFHF for 4 weeks compared to chow‐fed animals (−34 mg of glucose/dl in 30 min for the HFHF‐fed dogs vs. −48 mg/dl in 30 min in the control chow‐fed dogs), but the nadir of glucose and the secretion of glucagon and other counterregulatory hormones were the same during the rest of the experiment. The goal in the present study was to look at the impact of CHADN on the response exhibited by HFHF‐fed dogs.

Our previously published efficacy data from the animals indicate that, after CHADN, the glucose excursion in response to an OGTT was improved compared to the response in SHAM animals. This improvement was due to a change in insulin secretion and an improvement of the hepatic uptake of glucose during postprandial conditions (Kraft et al., 2019). To the extent that this was due to an improvement in insulin sensitivity, one could argue that, in the present study, the CHADN animals would be more prone to hypoglycemia in response to the same dose of insulin but this was not the case. The main question addressed here is whether CHADN predisposes to hypoglycemia when a set amount of insulin was infused. For the purpose of this study, a lack of safety would be characterized as a difference in the rate of glucose decrease, a change in the nadir of glucose, or a change in the counterregulatory response. Our data indicated that no such changes occurred, an important observation in support of the utility of CHADN.

Previously, we demonstrated that afferent nerves originating in the hepatoportal region, regardless of their path to the brain, are not necessary for a complete counterregulatory response to an insulin‐induced hypoglycemic challenge (Jackson et al., 2000). Other studies focused on the effect of liver transplant on hepatic glucose metabolism and the response to hypoglycemia (Colle et al., 2004). Even though liver transplant was reported to negatively affect insulin resistance, liver denervation associated with the transplant had no major deleterious effects on bile secretion, liver regeneration, or hepatic blood flow in humans. Numerous models of liver transplant showed that the counterregulatory response to insulin‐induced hypoglycemia or exercise (swimming and running) is normal (Jackson et al., 2000; Latour et al., 1985; Lindfeldt et al., 1993; Moore et al., 1993). Although increased hypoglycemic incidence does not appear to be a complication in those patients, there are numerous conflicting factors (such as the use of corticosteroids or immunosuppressive agents) which complicate interpretation of the data (Luzi et al., 1997; Moore et al., 2002). On the other hand, studies investigating the importance of brain hypoglycemia to the counterregulatory response using glucose infusion into the carotid and vertebral arteries (Biggers et al., 1989; Winnick et al., 2016) showed that the brain appeared to play a major role in coordinating the hormonal response to hypoglycemia. Other data suggest that the α cell response to hypoglycemia is dependent on pancreatic innervation (Berthoud et al., 1990; Biggers et al., 1989), and that hepatic glucose production depends on liver innervation during insulin‐induced hypoglycemia (Puschel, 2004). However, when islets or isolated α cells are studied, the secretion of glucagon is still under the control of glucose levels (Cryer, 2008; Munoz et al., 2005). In the case of liver transplant, both sympathetic and parasympathetic nervous systems are affected. Our study focuses the denervation process on the common hepatic artery, making sure that the vagal nerves stayed intact and the brain was exposed to hypoglycemia. By targeting the common hepatic artery, we only alter the sympathetic nervous system and not the parasympathetic nervous system that runs through the vagal nerve and the portal vein as was done in previous studies (Berthoud, 2004; Uyama et al., 2004). Cutting the sympathetic tone to the liver, and not the parasympathetic tone (i.e., vagal nerve), is key to the improvement of liver insulin sensitivity and glucose excursion observed during an OGTT (Kraft et al., 2019). The hypothesis that the sympathetic nervous system is overactive in those with obesity, insulin‐resistant states, and, in general, in the metabolic syndrome is not a novel idea (Lohmeier & Iliescu, 2013; Thorp & Schlaich, 2015). Modulation of sympathetic nerve activity may prove to be a means of permanently treating multiple conditions associated with the metabolic syndrome. As such, we are in agreement with colleagues working on renal sympathetic denervation. Multiple clinical trials are underway to test the effect of catheter‐based renal denervation in obese, drug‐resistant cases of hypertension (Mahfoud et al., 2011). These cohorts suggested that renal denervation is safe (Kandzari et al., 2018; Sanders et al., 2017). The primary safety end point was most often a composite of death, end‐stage renal disease, embolic events resulting in end‐organ damage, and renovascular complications. The data related to kidney function were not relevant in our study and we did not measure it, as there is no reason to believe that a denervation of the common hepatic artery would alter the kidney function. We could not see any increase in mortality (after 3 months) or in any factors measured monthly and reported in our bloodwork data. In addition, no organ defects were found on necropsy. The site of denervation around the common hepatic artery presented some scarring, but there was no defect in vessel wall or function. There were no visible effects of the surgery on day‐to‐day life of the animals. Contrary to Roux‐en‐Y gastric bypass that is also used to treat the metabolic syndrome and is in theory reversible, the CHADN procedure appears to be irreversible, with there being no sign of nerve regrowth for at least 3 months post‐surgery. It remains to be seen if our results (both efficacy Kraft et al., 2019 and safety presented in this paper) translate to the clinical setting when the procedure is performed using a catheter ablation to limit invasiveness of the technique.

Nonetheless, the present study demonstrates that surgical ablation of the common hepatic artery is safe in our diet‐induced glucose‐intolerant dog model and does not amplify the effect of exogenous insulin on hypoglycemia. Even if the mechanisms involved in the response to hypoglycemic are in part neurally mediated, the counterregulatory hormones are secreted in adequate quantities and with a time pattern similar in animals receiving the CHADN and those receiving a SHAM procedure.

## CONFLICTS OF INTEREST

B.R.A. was employee at the time of the study and hold equity in Metavention. A.D.C. received research funding from Metavention for this study, is a member of the Metavention Scientific Advisory Board, and holds stock options in Metavention. No other potential conflict of interest relevant to this article was reported.

## AUTHOR CONTRIBUTIONS

G.K. directed all experiments, collected and interpreted data, and drafted and revised the manuscript, D.S.E. and B.R.A. participated in the design of the experiment and reviewed the manuscript. M.S., E.A., and D.S.E. participated in the experiments and biochemical analysis of the samples. B.F. was responsible for surgical preparation and oversight of animal care. B.R.A. and A.D.C. interpreted the results, contributed to the discussion, and edited the manuscript. A.D.C. is the guarantor of this work and, as such, had full access to all the data in the study and takes responsibility for the integrity of the data and the accuracy of the data analysis.

## Supporting information



Fig S1

Table S1

Table S2
