# Supplementary material for: Safety of surgical denervation of the common hepatic artery in insulin‐resistant dogs
Source: Physiol Rep. 2021 Mar 26;9(6):e14805. doi: 10.14814/phy2.14805 (PMC7995543; doi:10.14814/phy2.14805)
Supplement: Supplementary file 1 — Fig S1 [file PHY2-9-e14805-s002.pdf]

Supplemental figure 1

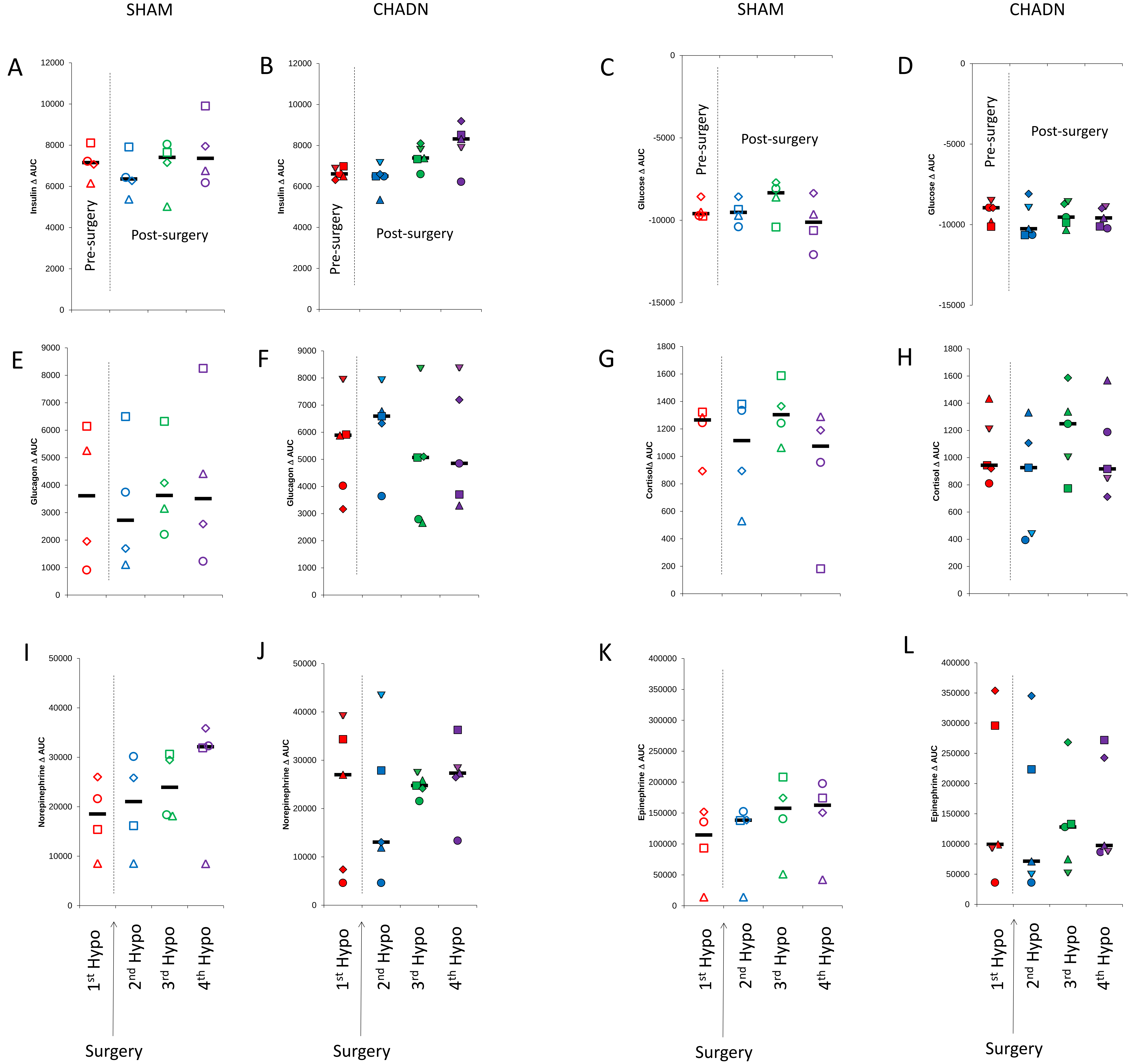

Delta Area under the curve (Δ AUC) for multiple parameters measured during the 3 hours of hypoglycemic challenge. Insulin (A and B in µU . 180 min / mL), glucose (C and D, in mg . 180 min / dL), glucagon (E and F, in pg . 180 min / dL), cortisol (G and H, in µg . 180 min / dL), norepinephrine (I and J, in pg . 180 min / dL) and epinephrine (K and L, in pg . 180 min / dL) in successive hypoglycemic studies. Each symbol represent one individual animal studied multiple times, the black bars represent the median of the group. Open symbols represent animals in the SHAM group, solid symbols represent animals in the CHADN group. The animals were studied 4 times, in red the 1stHypo study (before treatment, SHAM or CHADN), in blue the 2nd Hypo, in green the 3rd Hypo, in purple the 4th Hypo.
