# Supplementary material for: Safety of surgical denervation of the common hepatic artery in insulin‐resistant dogs
Source: Physiol Rep. 2021 Mar 26;9(6):e14805. doi: 10.14814/phy2.14805 (PMC7995543; doi:10.14814/phy2.14805)
Supplement: Supplementary file 2 — Table S1 [file PHY2-9-e14805-s003.pdf]

SUPPLEMENTAL TABLE 1 BLOODWORK RESULTS

CHADN DOGS
n=5

			High-Fat / High-Fructose Diet									
							CHADN					
		Week	0		5		9		13		17	
Units	Normal		Average	SEM	Average	SEM	Average	SEM	Average	SEM	Average	SEM
kg		Weight (kg)	23.3	0.6	25.4	1.0	26.4	0.9	26.8	1.1	27.0	1.0
		SUPERCHEM										
g/dL	5.0-7.4	Total Protein	6.2	0.12	5.9	0.13	6.0	0.10	6.2	0.16	6.1	0.14
g/dL	2.7-4.4	Albumin	3.6	0.08	3.4	0.08	3.5	0.07	3.7	0.09	3.6	0.10
g/dL	1.6-3.6	Globulin	2.6	0.13	2.5	0.11	2.5	0.14	2.5	0.12	2.6	0.07
Ratio	0.8-2.0	A/G Ratio	1.4	0.07	1.4	0.08	1.4	0.10	1.5	0.07	1.4	0.05
U/L	15-66	AST (SGOT)	26	1.3	30	2.1	27	1.5	30	1.0	27	1.4
U/L	12-118	ALT (SGPT)	29	3.5	25	2.7	26	2.1	23	2.6	26	2.3
U/L	5-131	Alk Phosphatase	48	5.7	43	4.1	38	3.9	39	3.4	36	3.4
U/L	1-12	GGTP	4.3	0.8	3.3	0.5	3.2	0.3	3.5	0.5	3.7	0.5
mg/dL	0.1-0.3	Total Bilirubin	0.17	0.02	0.10	0.00	0.10	0.00	0.10	0.00	0.12	0.02
mg/dL	6-31	Urea Nitrogen	17	1.3	14	1.1	15	1.3	14	0.5	14	0.5
mg/dL	0.5-1.6	Creatinine	0.98	0.07	0.85	0.04	0.90	0.06	0.88	0.05	0.93	0.05
Ratio	4-27	BUN/Creatinine Ratio	17	1.7	17	1.5	18	2.2	16	1.2	15	0.6
mg/dL	2.5-6.0	Phosphorus	5.3	0.2	5.3	0.2	5.1	0.2	4.8	0.1	4.5	0.2
mg/dL	70-138	Glucose	100	2.2	98	2.9	96	3.5	98	2.7	96	2.4
mg/dL	8.9-11.4	Calcium	10.7	0.16	10.6	0.07	10.5	0.06	10.6	0.19	10.5	0.12
mEq/L	1.5-2.5	Magnesium	1.50	0.04	1.45	0.02	1.48	0.07	1.47	0.04	1.47	0.02
mEq/L	139-154	Sodium	147	0.6	146	0.4	146	0.5	146	0.7	146	0.7
mEq/L	3.6-5.5	Potassium	4.6	0.09	4.6	0.02	4.9	0.07	4.9	0.11	4.6	0.08
		Na/K Ratio	31.5	0.62	32.0	0.00	30.0	0.40	30.2	0.72	31.7	0.61
mEq/L	102-120	Chloride	113	0.6	111	0.8	111	0.8	110	0.8	112	0.5
mg/dL	92-324	Cholesterol	171	9.0	214	10.6	230	15.2	244	10.1	226	6.5
mg/dL	29-291	TG	37	4.6	39	3.4	43	4.9	43	5.0	41	2.6
U/L	290-1125	Amylase	651	33.0	612	73.5	648	34.4	664	33.8	633	27.2
U/L	77-695	Lipase	227	41.1	204	32.8	250	50.3	262	54.6	254	43.2
U/L	59-895	CPK	182	17.9	181	18.4	165	17.8	191	6.2	157	15.9
		CBC										
x10(3) / µl	4.0 - 15.5	WBC	8.5	0.7	9.8	1.0	9.1	0.4	9.3	0.4	9.4	0.4
x10~6c / µl	4.8 - 9.3	RBC	6.5	0.2	6.6	0.1	7.1	0.2	7.1	0.2	7.0	0.2
g/dl	12 - 20	HGB	15.4	0.6	15.6	0.4	16.7	0.3	16.8	0.5	16.3	0.4
%	36 - 60	HCT	47.2	1.5	47.3	0.9	51.7	1.4	51.0	1.3	51.7	1.6
fL	58 - 79	MCV	72.7	1.1	71.8	0.5	73.0	0.9	72.0	0.6	73.8	0.8
pg	19 - 28	MCH	23.8	0.5	23.7	0.4	23.7	0.2	23.8	0.4	23.3	0.1
g/dL	30 - 38	MCHC	32.7	0.7	32.9	0.4	32.4	0.5	32.9	0.4	31.6	0.4
x10^3 / µl	170-400	PLT Count	220	23	243	46	249	24	242	21	258	32
		PLT EST	ad		ad		ad		ad		ad	
		WBC Differential										
/ µl	2060-10600	Neutrophils	4633	457	5730	644	5101	225	5359	213	5465	124
/ µl	0-300	Bands	0	0	0	0	0	0	0	0	0	0
/ µl	690-4500	Lymphocytes	2754	263	2889	276	3146	198	2901	229	2942	216
/ µl	0-840	Monocytes	442	40	575	100	364	36	389	43	375	25
/ µl	0-1200	Eosinophils	581	148	560	159	493	143	588	128	583	178
/ µl	0-150	Basophils	40	20	30	21	31	21	48	24	49	24
