# Supplementary material for: Safety of surgical denervation of the common hepatic artery in insulin‐resistant dogs
Source: Physiol Rep. 2021 Mar 26;9(6):e14805. doi: 10.14814/phy2.14805 (PMC7995543; doi:10.14814/phy2.14805)
Supplement: Supplementary file 3 — Table S2 [file PHY2-9-e14805-s001.pdf]

SUPPLEMENTAL TABLE 2 BLOODWORK RESULTS

SHAM DOGS
n=4

High-Fat / High-Fructose Diet

SHAM

		Week	0		5		9		13		17	
Units	Normal		average	SEM	average	SEM	average	SEM	average	SEM	average	SEM
kg		Weight (kg)	22.5	0.9	24.1	0.8	25.4	0.9	25.4	0.9	25.8	1.1
		SUPERCHEM										
g/dL	5.0-7.4	Total Protein	5.9	0.21	5.9	0.17	5.8	0.17	5.8	0.13	5.8	0.10
g/dL	2.7-4.4	Albumin	3.6	0.07	3.5	0.07	3.4	0.07	3.6	0.03	3.4	0.09
g/dL	1.6-3.6	Globulin	2.4	0.14	2.4	0.13	2.4	0.10	2.2	0.12	2.4	0.07
Ratio	0.8-2.0	A/G Ratio	1.5	0.08	1.5	0.07	1.5	0.03	1.7	0.09	1.5	0.06
U/L	15-66	AST (SGOT)	23	1.2	27	1.3	30	1.9	33	1.0	28	2.9
U/L	12-118	ALT (SGPT)	31	3.7	24	3.2	24	2.7	25	2.9	25	1.9
U/L	5-131	Alk Phosphatase	51	16.5	43	13.4	42	14.4	44	16.7	39	14.1
U/L	1-12	GGTP	4.3	0.6	3.0	0.8	3.3	0.3	4.0	0.5	3.8	0.3
mg/dL	0.1-0.3	Total Bilirubin	0.13	0.03	0.13	0.03	0.10	0.00	0.10	0.00	0.10	0.00
mg/dL	6-31	Urea Nitrogen	17	1.4	14	0.7	14	0.7	13	1.3	14	0.5
mg/dL	0.5-1.6	Creatinine	0.83	0.03	0.83	0.03	0.83	0.03	0.88	0.03	0.90	0.05
Ratio	4-27	BUN/Creatinine Ratio	20	1.5	17	1.5	18	1.1	15	1.4	16	1.4
mg/dL	2.5-6.0	Phosphorus	5.2	0.1	4.8	0.3	4.0	0.7	4.1	0.4	4.2	0.2
mg/dL	70-138	Glucose	101	4.1	97	6.7	97	3.6	101	6.5	93	2.6
mg/dL	8.9-11.4	Calcium	10.1	0.13	10.2	0.03	10.3	0.09	10.1	0.28	10.1	0.26
mEq/L	1.5-2.5	Magnesium	1.63	0.07	1.50	0.05	1.53	0.11	1.48	0.06	1.50	0.07
mEq/L	139-154	Sodium	148	1.5	146	1.2	146	0.6	147	1.2	145	0.7
mEq/L	3.6-5.5	Potassium	4.4	0.14	4.6	0.11	5.0	0.07	4.8	0.17	4.7	0.14
		Na/K Ratio	34.3	0.99	32.0	0.82	29.8	0.55	30.5	1.00	30.8	0.73
mEq/L	102-120	Chloride	113	1.7	112	1.0	112	0.9	113	0.9	113	0.9
mg/dL	92-324	Cholesterol	158	28.1	217	27.2	218	23.5	233	33.3	222	31.7
mg/dL	29-291	TG	40	12.6	35	3.7	49	19.2	40	6.0	35	2.4
U/L	290-1125	Amylase	584	18.9	760	103.5	773	83.3	724	90.9	646	59.4
U/L	77-695	Lipase	177	19.4	212	64.3	248	45.2	270	73.4	228	23.8
U/L	59-895	CPK	149	14.7	143	15.4	148	18.4	224	34.0	162	19.6
		CBC										
x10(3) / µl	4.0 - 15.5	WBC	8.7	1.2	10.2	1.0	9.1	0.5	9.1	1.0	8.9	0.8
x10~6c / µl	4.8 - 9.3	RBC	6.4	0.4	6.5	0.3	6.8	0.1	7.1	0.3	6.7	0.2
g/dl	12 - 20	HGB	15.7	0.9	15.4	0.7	15.9	0.4	17.0	0.6	15.8	0.3
%	36 - 60	HCT	46.5	2.8	47.8	1.0	49.0	0.6	52.0	1.1	49.8	1.3
fL	58 - 79	MCV	72.5	1.0	73.3	1.8	72.3	1.2	73.3	1.7	74.5	1.9
pg	19 - 28	MCH	24.4	0.5	23.6	0.3	23.4	0.2	23.9	0.2	23.7	0.3
g/dL	30 - 38	MCHC	33.7	0.2	32.3	0.8	32.5	0.5	32.6	0.5	31.9	1.1
x10^3 / µl	170-400	PLT Count	225	25	269	26	258	12	266	26	215	34
		PLT EST	ad		ad		ad		ad		ad	
		WBC Differential										
/ µl	2060-10600	Neutrophils	4583	870	5944	676	5119	655	5239	748	5035	750
/ µl	0-300	Bands	0	0	0	0	0	0	0	0	0	0
/ µl	690-4500	Lymphocytes	3146	366	3223	337	2951	162	2981	223	2969	66
/ µl	0-840	Monocytes	563	38	500	66	429	129	377	38	430	88
/ µl	0-1200	Eosinophils	349	60	465	93	482	138	434	99	446	148
/ µl	0-150	Basophils	35	23	44	29	85	46	20	23	20	23
